# Concomitant mortality trends due to obesity and hypertension in the U.S.: a 20-year retrospective analysis of the CDC WONDER database

**DOI:** 10.1186/s12872-025-04909-z

**Published:** 2025-07-07

**Authors:** Rayyan Nabi, Alina Zanub, Muzamil Akhtar, Sohaib Aftab Ahmad Chaudhry, Abdul Raffay Awais, Hanzala Ahmed Farooqi, Sabahat Ul Ain Munir Abbasi, Peter Collins, Tabeer Zahid, Raheel Ahmed, Muhammad Zia Ur Rehman

**Affiliations:** 1 Islamic International Medical College, Rawalpindi, Punjab Pakistan; 2 Foundation University Medical College, Islamabad, Pakistan; 3 Gujranwala Medical College, Gujranwala, Punjab Pakistan; 4 ABWA Medical College, Faisalabad, Pakistan; 5 Allama Iqbal Medical College, Lahore, Pakistan; 6 National Heart and Lung Institute, Imperial college London, Sydney Street, London, SW3 6NP UK

**Keywords:** Obesity, Hypertension, CDC WONDER, Age adjusted mortality rate, Retrospective analysis

## Abstract

**Background:**

Hypertension (HTN) and obesity are leading, interrelated risk factors for cardiovascular disease, stroke, and kidney disease in the United States. Despite advances in medical therapies and public health interventions, the joint mortality burden associated with these conditions remains substantial. We sought to characterize national trends and demographic disparities in obesity- and hypertension-related mortality from 2000 to 2019 using the CDC WONDER database.

**Methods:**

In this retrospective descriptive study, multiple cause-of-death data for individuals aged ≥ 25 years were extracted from CDC WONDER. Obesity (ICD-10 E66.*) and hypertension (ICD-10 I10–I15)-related deaths were identified as underlying or contributing causes. Crude Mortality Rates (CMRs) and Age-Adjusted Mortality Rates (AAMRs) per 100,000 population were calculated annually and standardized to the 2000 U.S. population. Joinpoint regression was employed to estimate Annual Percent Change (APC) and Average Annual Percent Change (AAPC) in AAMRs, with statistical significance set at *p* < 0.05. Trends were stratified by sex, race/ethnicity, urban-rural classification, and U.S. Census region.

**Results:**

From 2000 to 2019, there were 254,116 obesity- and hypertension-related deaths (54.6% male). The combined AAMR rose from 2.58 to 9.62 per 100,000 (AAPC 7.31*, 95% CI 6.66–7.97). Men experienced higher AAMRs than women (AAPC 8.50* vs. 6.08*, respectively). Non-Hispanic Black individuals exhibited the highest AAMR (11.19), followed by American Indian/Alaska Native (6.62) and non-Hispanic White (5.35) populations. Non-metropolitan counties demonstrated greater mortality (AAMR 6.52) compared to metropolitan areas (5.76), and Southern and rural states bore the highest burdens.

**Conclusions:**

Over two decades, obesity- and hypertension-related mortality in U.S. adults has increased significantly across all demographic groups, with pronounced disparities by sex, race/ethnicity, and rurality. Targeted, multifaceted interventions are urgently needed to curb this growing public health crisis.

**Supplementary Information:**

The online version contains supplementary material available at 10.1186/s12872-025-04909-z.

## Introduction

Hypertension (HTN) and obesity are leading risk factors for cardiovascular disease (CVD), stroke, and kidney disease [1–3]. The occurrence of obesity and hypertension in individuals involves a complex interplay which includes interactions between renal, metabolic, and neuroendocrine pathways [4]. While, obesity is known to cause approximately 65–78% of cases of hypertension [4] hypertension itself is also considered a leading cause of morbidity and mortality globally, with an estimated 10.4 million deaths annually [4]. Moreover, obesity is associated with higher mortality rates when coupled with co-morbidities such as hypertension [5]. This existing relationship between the two signifies the global impact that they possess. The prevalence of both conditions has risen globally, with significant impact in the U.S. Their combined burden has led to increased morbidity, healthcare costs, and premature mortality [4]. Over the past two decades, the prevalence of both conditions has risen due to changing dietary habits, sedentary lifestyles, and socioeconomic disparities [6, 7]. While advances in antihypertensive therapies and public health interventions have improved disease management, the mortality burden associated with hypertension and obesity remains substantial [8]. Understanding concomitant trends in mortality due to these conditions is crucial for assessing the effectiveness of existing prevention and treatment strategies, identifying disparities across demographic groups, and informing future healthcare policies. This study aims to analyze concomitant national trends in hypertension- and obesity-related mortality in the U.S. from 2000 to 2019, with a focus on variations by sex, race/ethnicity, urbanization and geographic region. By examining Age-Adjusted Mortality Rates (AAMRs), this analysis will provide insights into whether improvements in hypertension management have mitigated the mortality impact of rising obesity rates or whether disparities persist among vulnerable populations.

## Methods

### Study setting and population

In this retrospective descriptive study, mortality data related to obesity and hypertension were retrieved from the Centers for Disease Control and Prevention Wide-Ranging Online Data for Epidemiologic Research (CDC WONDER) database. The CDC WONDER database provides publicly available national mortality data from death certificates collected by the National Center for Health Statistics (NCHS). We used the Multiple Cause of Death files, which include data from all 50 U.S. states and the District of Columbia about both underlying and contributing causes of death, along with geographic and demographic variables such as age (grouped from < 1 year to 85+), sex (male/female), race and ethnicity. Data from 2000 to 2019 were examined to assess trends in mortality associated with obesity and hypertension. Although available, data from 1999 was considered unreliable for the Non-Hispanic American Indian or Alaska Native population, while data from 2020 was excluded to avoid potential bias related to the impact of the COVID-19 pandemic. The following International Classification of Diseases, 10 th Revision, Clinical Modification (ICD-10) codes were used to identify obesity (E66.0, E66.1, E66.2, E66.8, E66.9) [9] and hypertension (I10 - I15**)** [10]. These are codes that have been validated for identifying obesity and hypertension-related mortality in previous epidemiological studies [9, 10]. Obesity and hypertension-related deaths were identified based on their appearance as either contributing or underlying causes on the death certificates. This study was exempt from institutional review board approval, as it used deidentified, publicly available government data, and adhered to the Strengthening the Reporting of Observational Studies in Epidemiology (STROBE) guidelines for reporting [11].

### Data abstraction

Data for the study were abstracted from the CDC WONDER database including population size, year, urban-rural classification, regional classification, gender, and race. Only individuals aged 25 years or older were included in the analysis. Race categories were defined as Hispanic, non-Hispanic (NH) White, NH Black, NH Asian or Pacific Islander, and NH American Indian or Alaska Native. The National Center for Health Statistics Urban-Rural Classification Scheme was used to categorize counties by urban (large metropolitan area (population ≥ 1 million), medium/small metropolitan area (population 50,000–999,999) and rural areas (population < 50,000) [12]. Regions were classified into 4 groups; Northeast, Midwest, South, and West according to the U.S Census Bureau Definitions [12].

### Statistical analysis

Crude Mortality Rates (CMRs) and Age-Adjusted Mortality Rates (AAMRs) per 100,000 populations were calculated for obesity and hypertension-related mortality from 2000 to 2019. Mortality rates were determined by dividing the number of deaths related to obesity or hypertension by the corresponding U.S. population for each year. AAMRs were calculated by standardizing the mortality data to the year 2000 U.S. population; this method is widely used as it permits the comparison of mortality rates while adjusting for age differences in the population. To analyze national trends in obesity and hypertension-related mortality, Joint-point Regression Program (Joint point V 4.9.0.0, National Cancer Institute) was used to determine the Annual Percent Change (APC) in AAMR over the study period [13]. Log-linear regression models were used to identify significant temporal changes in mortality rates, and APCs were considered increasing or decreasing if the slope of the regression line was significantly different from zero. Joint point regression was applied to identify inflection points in temporal trends, the guidelines state that in datasets with time-points in the range of 17 to 21, it is recommended to identify a maximum of three inflection points - this study had 20 time-points. Thus, the joint point regression software was set to identify up to 4 join points, however, fewer than this could be identified if the magnitude of variation between trends was greatest with fewer inflection points. The Grid Search method (0, 2), combined with a permutation test and parametric method, was used to estimate the APC and corresponding 95% confidence interval (CI). APC describes the rate of change of AAMR over time, providing a measure of how mortality rates vary annually. If it is positive, it shows an increase, whereas a negative APC reflects a decrease in mortality rates. A two-tailed t-test was used for statistical significance of APC values, with *p* < 0.05 considered statistically significant.

## Results

From 1999 to 2019, there were a total of 254,116 deaths due to obesity and hypertension (HT) in the US population aged 25 years to over 85 years. Of these 138,611 occurred in males while 115,505 occurred in females. The results were categorized by year, gender, race, urbanization, and state.

### Obesity related AAMR

**Fig. 1:**
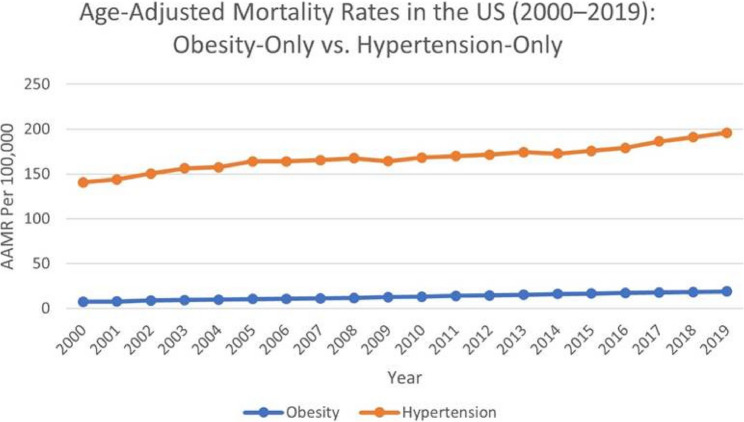
Age–adjusted mortality rates in the US (2000–2019): Obesity–Only vs. Hypertension–Only

AAMR (age-adjusted mortality rate) attributable to obesity surged sharply between 2000 and 2003 at APC (Annual Percentage Change) of (APC 9.51*; 95% CI: 3.68–15.67), marking a significant early escalation. Thereafter, rates continued their significant ascent at a steadier pace throughout 2003– 2019 (APC 3.21*; 95% CI: 2.88–3.54). Over the entire 2000–2019 window, the burden increased consistently at AAPC (Average Annual Percentage Change) of (AAPC 4.18*; 95% CI: 3.32–5.05), underscoring a persistent upward trajectory (Fig. 1).

### Hypertension related AAMR

The age–adjusted mortality rate (AAMR) linked to hypertension climbed modestly from 2000 to 2003 (APC 4.69*; 95% CI: 2.18–7.27), a statistically significant rise. The trend then flattened during 2003– 2011 (APC 0.34; 95% CI: − 0.25–0.92) and remained statistically non–significant in 2011– 2014 (APC 1.90; 95% CI: − 2.12–6.09). A renewed, more pronounced increase appeared in 2014– 2019 (APC 4.66*; 95% CI: 3.83–5.50), again significant. Across the full 2000– 2019 interval, mortality rose overall (AAPC 2.39*; 95% CI: 1.67–3.11), confirming a sustained upward pattern (Fig. 1).

### Overall obesity and HT related AAMR

The age–adjusted mortality rate (AAMR) attributable to obesity and hypertension rose sharply from 2000 to 2003 (APC 10.80*; 95% CI: 6.73–15.03), marking a significant trend. This significant growth continued during 2003–2015 (APC 7.28*; 95% CI: 6.91–7.66) and persisted through 2015–2019 (APC 4.84*; 95% CI: 3.61–6.09). Across the entire 1999–2019 interval, the AAMR increased from 2.58 in 1999 to 9.62 in 2019, maintaining a significant upward trajectory (AAPC 7.31*; 95% CI: 6.66–7.97).

From 1999 to 2019, the overall AAMR was higher in males (6.77) than in females (5.00). Among males, mortality surged significantly between 2000 and 2003 (APC 12.30*; 95% CI: 8.21–16.53). The significant rise persisted through 2003–2014 (APC 8.57*; 95% CI: 8.15–8.99) and continued, though at a slightly slower pace, from 2014 to 2019 (APC 6.13*; 95% CI: 5.33–6.94). Overall, the 2000–2019 period showed a sustained significant increase (AAPC 8.50*; 95% CI: 7.86–9.14).

**Fig. 2:**
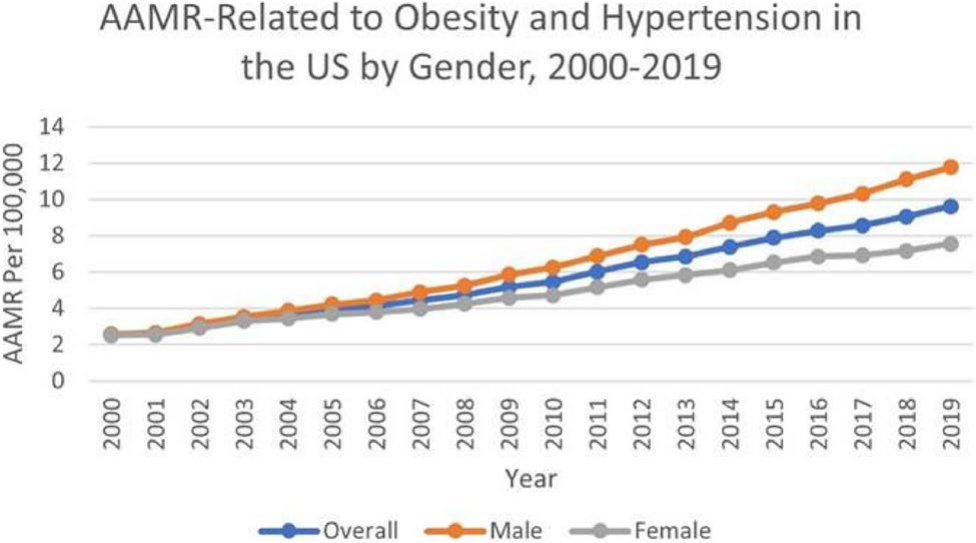
AAMR-related to obesity and hypertension in the US by Gender, 2000–2019

For females, the AAMR climbed significantly from 2000 to 2003 (APC 9.52*; 95% CI: 4.95–14.28). This significant upward trend continued during 2003–2015 (APC 6.08*; 95% CI: 5.64–6.53) and remained significant from 2015 to 2019 (APC 3.54*; 95% CI: 2.02–5.10). Over the entire 2000–2019 span, female mortality displayed a significant overall increase (AAPC 6.08*; 95% CI: 5.33–6.83) (Fig. 2).

### Obesity and HT related AAMR stratified by race/ethnicity

**Fig. 3:**
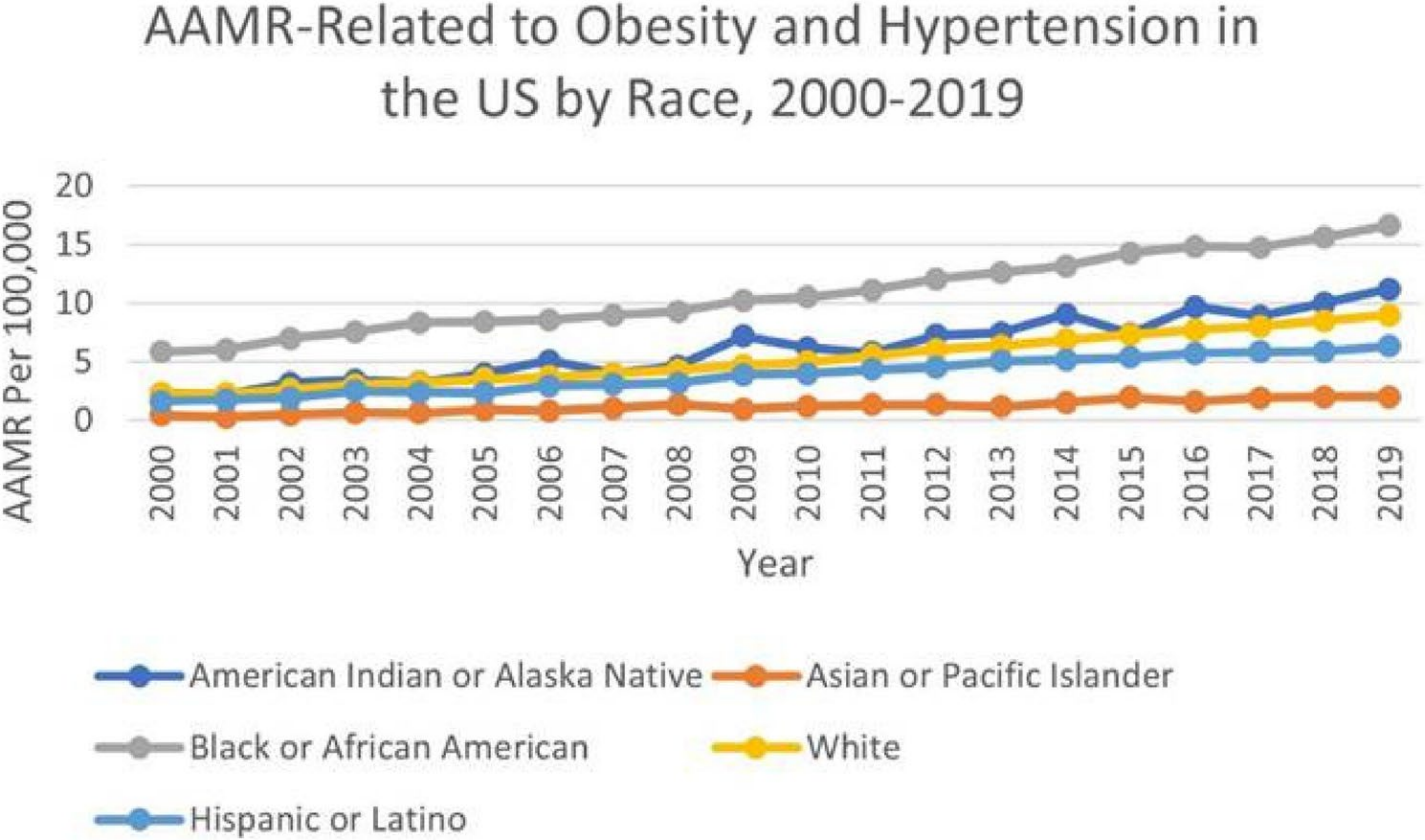
AAMR-related to obesity and hypertension in the US by Race, 2000–2019

From 1999 to 2019, Black or African American individuals exhibited the highest overall AAMR (11.19), followed by American Indian or Alaska Native individuals (6.62), White individuals (5.35), Hispanic or Latino individuals (4.29), and with the lowest AAMR in Asian or Pacific Islander individuals (1.33). In Black or African American, mortality rose sharply in 2000–2004 (APC 9.58*; 95% CI: 6.11–13.15), marking a significant surge. The trend then flattened during 2004–2007 (APC 2.48; 95% CI: − 5.88–11.58), not reaching significance. Growth resumed between 2007 and 2015 (APC 5.93*; 95% CI: 4.96–6.92) and persisted from 2015 to 2019 (APC 3.75*; 95% CI: 1.98–5.56); both intervals reflect significant increases. Overall, 2000–2019 showed a sustained rise (AAPC 5.67*; 95% CI: 4.22–7.14). In American Indian or Alaska Native individuals, across 2000–2019, the AAMR climbed in a single, uninterrupted upswing (APC 7.54*; 95% CI: 6.32–8.76), indicating a significant and steady rise. The Average Annual Percent Change mirrored this value (AAPC 7.54*; 95% CI: 6.32–8.76). For White individuals, mortality climbed significantly from 2000 to 2013 (APC 8.33*; 95% CI: 7.90–8.77) before slowing to a still significant rate in 2013–2019 (APC 5.64*; 95% CI: 4.78–6.52). Collectively, the study window shows a consistent increase (AAPC 7.48*; 95% CI: 7.11–7.85). In Hispanic or Latino individuals, AAMR increased markedly between 2000 and 2013 (APC 8.65*; 95% CI: 7.68–9.63), underscoring a significant rise, and continued upward during 2013–2019 (APC 3.59*; 95% CI: 2.00–5.21), also significant. Across 2000–2019, mortality increased overall (AAPC 7.03*; 95% CI: 6.26–7.80). For Asian or Pacific Islander individuals, a continuous increase was observed from 2000 to 2019 (APC 7.45*; 95% CI: 5.95–8.98), denoting a significant upward trend throughout the period; the AAPC was identical (7.45*; 95% CI: 5.95–8.98) (Fig. 3).

### Obesity and HT related AAMR stratified by urbanization status

From 1999 to 2019, a higher overall AAMR was observed in non-metropolitan areas (6.52) as compared to metropolitan areas (5.76). In non–metropolitan counties, mortality surged sharply between 2000 and 2004 (APC 11.40*; 95% CI: 6.83–16.16), marking a significant early escalation. Although the tempo eased thereafter, rates continued their significant ascent from 2004 to 2019 (APC 7.52*; 95% CI: 7.14–7.91). Across the entire study window, the burden increased overall at a significant pace (AAPC 8.33*; 95% CI: 7.41–9.25).

**Fig. 4:**
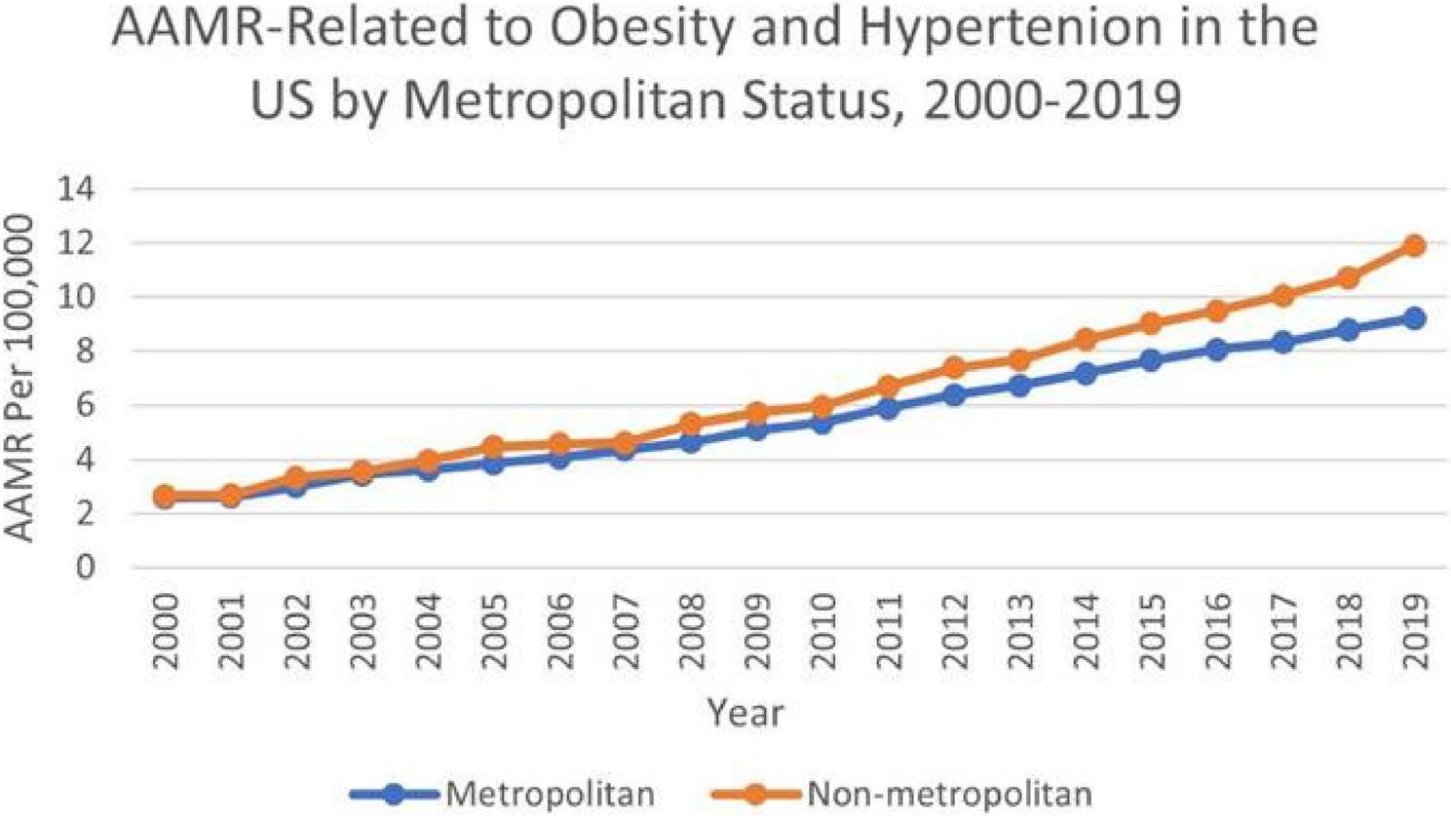
AAMR-related to obesity and hypertension in the US by Urbanization, 2000–2019

The age–adjusted mortality rate (AAMR) for obesity and hypertension in metropolitan counties rose briskly from 2000 to 2014 (APC 7.57*; 95% CI: 7.23–7.92), a significant climb. Growth persisted, though at a more moderate pace, during 2014– 2019 (APC 4.78*; 95% CI: 3.75–5.82), again registering a significant increase. Taken together, the full 2000–2019 span shows a sustained and significant elevation in mortality (AAPC 6.83*; 95% CI: 6.48–7.18) (Fig. 4).

### Obesity and HT AAMR stratified by state

**Fig. 5:**
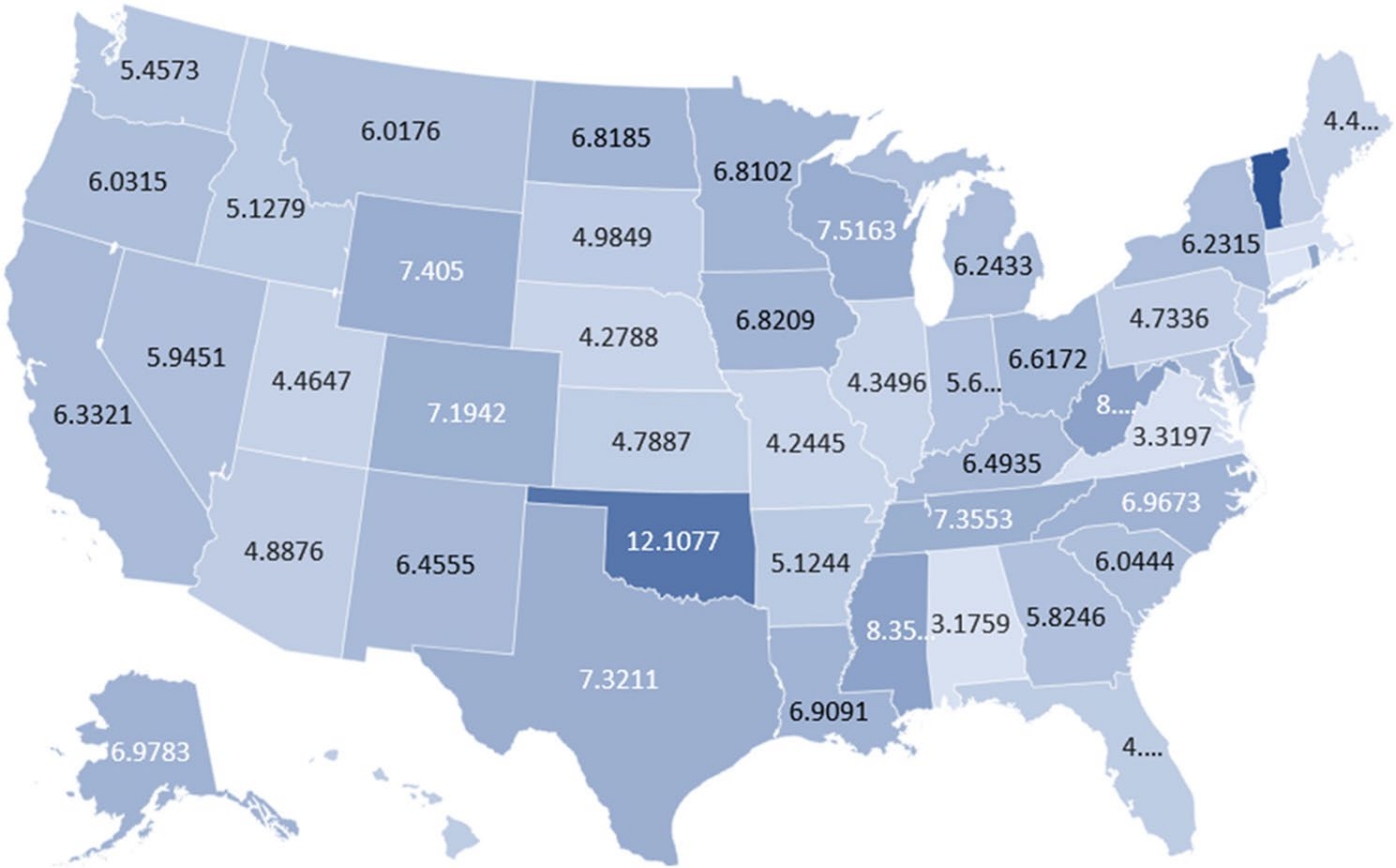
AAMR-related to obesity and hypertension in the US by States, 2000–2019

Between 1999 and 1999, the highest AAMR was observed in Vermont (AAMR 14.82; 95% CI 14.06 to 15.59), followed by Oklahoma (12.11; 95% CI 11.80 to 12.41), the District of Columbia (11.02; 95% CI 10.28 to 11.75), West Virginia (8.39; 95% CI 8.04 to 8.74), and Mississippi (8.35; 95% CI 8.06 to 8.64) appear at the upper end of the distribution. By contrast, Connecticut registered the lowest rate at 3.01 (95% CI 2.86 to 3.16), while Alabama 3.18 (95% CI 3.04 to 3.31), Virginia 3.32 (95% CI 3.21 to 3.43), Massachusetts 3.42 (95% CI 3.31 to 3.54), and Missouri 4.24 (95% CI 4.11 to 4.38) also lie toward the lower range (Fig. 5).

**Fig. 6:**
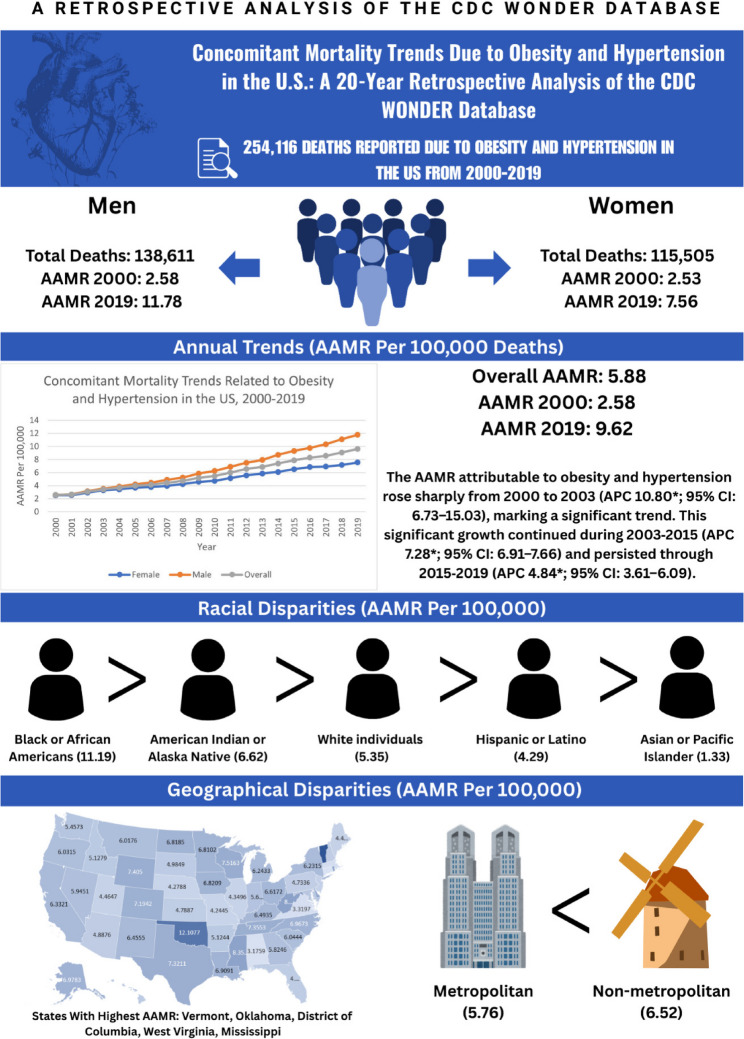
Central illustration

A summary of our results can be seen in the central illustration (Fig. 6).

## Discussion

Despite substantial efforts, hypertension and obesity remain foremost contributors of morbidity and mortality in the United States. Through the use of anonymized, age-adjusted data from the CDC WONDER database, our study sought to investigate patterns of this growing epidemic, with specific insights into how this burgeoning crisis has impacted diverse segments of the population.

Broadly, a generalized rise in age-adjusted mortality rates attributed to both obesity and hypertension were observed between 2000 and 2019 in all subsets of the population— whether stratified by sex, race, or regional differences. Though mortality rates overall reflect a sustained upward trajectory, demonstrated by higher mortality observed towards the end of the study as compared to the initial period, it is pertinent to mention that the rate of increase has progressively slowed overtime, reflected by reduced annual percentage change. Similarly, though fluctuations are observed when analyzing trends for obesity and hypertension as independent disease entities, the overarching pattern persists; mortality for either condition has significantly risen throughout the study period. It is noteworthy to mention, however, that while hypertension-related mortality demonstrates a decline in annual percentage change when comparing rates from 2000 to 2003 vs. 2003—2011, a curious acceleration was observed after 2014, which continued until the end of the study period.

Additionally, throughout the study period males consistently demonstrated higher mortality rates attributed to both diseases compared to their female counterparts, despite both groups exhibiting decelerating annual percentage changes. This was also true of race stratified trends, with mortality rates remaining highest among the Non-Hispanic Black population, followed by Non-Hispanic American Indians, Non-Hispanic Whites, Hispanics and finally Non-Hispanic Asians. Regional variations showed a similar pattern; non-metropolitan regions of the country experienced higher mortality rates. In a similar vein, we observed states composed of predominantly rural populations, such as Vermont, and Southern states with considerable rural populations, including Oklahoma, West Virginia, and Mississippi exhibited some of the highest mortality rates in the country. In contrast, states composed of largely urban populations generally exhibited lower mortality rates, regardless of region, including Southern states like Alabama and Virginia, as well as other states like Connecticut, Massachusetts and Missouri.

Rising age-adjusted mortality rates have been documented in previously published studies that have analyzed mortality trends using the CDC WONDER database in hypertension [14] and obesity [15, 16] independently, echoing similar patterns across demographic stratifications. Increasingly, literature suggests that obesity in America and hypertension are perhaps not two separate epidemics, rather a conglomeration of a shared pathology [17]. Such a relationship between the two conditions was first recognized in the Framingham Heart Study, which documented obese individuals have higher relative risks of developing comorbid hypertension [18]. Perhaps this may be due to overlapping etiopathogenic factors that contribute to the development and progression of both hypertension and obesity, including systemic low-grade inflammation leading to the metabolic syndrome [19] physical inactivity, and processed diets high in fats and sodium [20]. Other researchers have suggested obesity itself may be a contributory factor in developing hypertension [17, 21, 22] implicating factors such as insulin and leptin mediated stimulation of the sympathetic nervous system, and subsequent activation of renin-angiotensin-aldosterone system [17, 23] as well as micro-vascular damage and endothelial dysfunction associated with increased peripheral vascular resistance [24]. Furthermore, co-existence of the two morbidities appears to have a synergistic impact on adverse health outcomes including cardiovascular diseases [25] and renal injury [26, 27]. Hence, it may be hypothesized worsening morbidity in one condition can exacerbate the other, leading to overall greater mortality when considering both diseases. This concept is illustrated by our observation that the annual percentage change in mortality attributed to concomitant obesity and hypertension is consistently higher than the annual percentage change of either disease when considered as independent entities across the entire study period. Conceivably, the enhanced rates may perhaps have been the results of synergistic effects of the two compounding and catalyzing adverse outcomes thus leading to higher observed mortality when considered together.

Yet, despite increased awareness regarding risk factors and advances in treatment, the disease burden continues to rise [28–32]. Extensive literature argues rising nationwide obesity rates are largely driven by shifts in American lifestyles, e.g. reliance on ultra-processed foods due to lower cost and availability, coupled with sedentary living [33–40]. Moreover, barriers in the primary care setting prevent adequate treatment, including lack of insurance coverage, poor adherence to medication and lifestyle modifications, as well as inconsistent treatment counseling offered by physicians who typically stigmatize the condition; [41, 42]; barriers which are experienced disproportionately higher among minority groups [43]. In a similar vein, Munter et al., reports the age-adjusted estimated proportion of adults with controlled blood pressure decreased from 53.8% in 2013–2014, to 43.7% between 2017 and 2018 [44]. Perhaps this might have been due to new guidelines issued by the JNC-8 panel in 2013, which lowered blood pressure goals, hence de-intensified anti-hypertensive drug therapy. However, in 2017 the American Heart Association introduced new guidelines, replacing the JNC-8 in clinical practice [45]. These guidelines lowered the threshold for diagnosis and were predicted to increase the prevalence of adults defined as hypertensive, as well intensify drug therapy for those on antihypertensive medication [44]. Many studies anticipated the new guidelines would largely reduce mortality related to hypertension, as millions of adults would be recommended lifestyle changes, reduction in body weight and adoption of the DASH diet, and pharmacological therapy would be intensified in other groups [46]. Yet, the WHO still reports only 42% of adults with hypertension are diagnosed and treated, and only 21% have their blood pressure under control [46]. One study proposes that while theoretically such guidelines are projected to reduce morbidity and mortality, a problem remains in implementation [47]. Adequate counseling cannot be achieved in those who do not seek medical attention, and in those who can be counseled, it is difficult to implement such lifestyle changes in areas of food deserts or that lack walkable communities— the same factors which exacerbate obesity, a known risk factor for hypertension. Furthermore, medication non-adherence, affecting at least 40—50% of patients with hypertension [48] continues to pose a significant barrier management, serving as an independent risk factor for mortality [49]. Thus together, these findings underscore the complex interaction between biology, environment and behavior on higher observed mortality rates.

Notably, while both men and women experienced higher mortality rates due to obesity and hypertension throughout the study period, mortality rates in men remained consistently higher, a disparity that only grew with time. These findings have been replicated in research analyzing trends in obesity and hypertension independently, reporting worsening mortality especially in men [50]. Historically, obesity and hypertension tend to be deadlier diseases in men. It is well established in previous literature that due to differences in accumulation of visceral fat and lack of protection via estrogen, men suffer higher mortality due to obesity [51]. Similarly due differences in hormonal environments, men tend to suffer from higher rates of hypertension [52] but are less likely to be aware about their hypertensive status, hence resulting in poor control [53]. Furthermore, research has found that whether men suffer from obesity [54] or hypertension [53] they are less likely to implement beneficial lifestyle changes, engage health-seeking behaviors, and more often delay in seeking healthcare as compared to women. Adoption of such maladaptive behaviors, coupled with the burden of concurrent obesity and hypertension, thus may be responsible for the disproportionate mortality rates in men.

Interestingly, while women do suffer decreased mortality as compared to men, rising prevalence in women’s obesity rates have been increasingly recognized in literature. Between 2017 and 2018, the National Health and Nutrition Examination Survey reported that while the percentage of men with obesity is greater than the percentage of women, the percentage of women who have severe obesity is greater than the percentage of men with severe obesity. Furthermore, in adults 20 and over, there are no significant differences in prevalence of obesity by sex or age group [30]. Rising patterns in women’s obesity, while they do not contribute as immediately to higher mortality, do contribute to overall morbidity; Muenning et al. estimates that obesity obese women suffered a loss of 1.78 million quality adjusted life years, compared to obese men, who only lost around 270,000 [53]. Hence, it is imperative that women’s health not be overlooked in the obesity crisis.

Notably, throughout the study period, Non-Hispanic Blacks continuously experienced the highest mortality rates due to obesity and hypertension. Munter et al., in a study documenting trends in hypertension control between 1999 and 2018, found hypertension seems to be more prevalent and less controlled among Non-Hispanic Black adults [55]. This finding has been corroborated by several other studies, noting that while indeed, blood pressure control has generally worsened in the United States in the last decade, significantly worsened control rates have been observed among racial minorities, especially in Non-Hispanic Black individuals [56]. In a similar vein, Black populations have been found to experience the highest prevalence of obesity rates in the United States [15, 57]. Some studies have implicated cultural factors, stating poor weight control among African American communities may be due to preferences for larger bodies [22] while others implicate the southern diet for higher rates of obesity and hypertension in Black populations [58]. Furthermore, literature cites that marginalized groups tend to face higher barriers when it comes to engaging in lifestyle modifications; one study reports obesogenic food environments tend to be disproportionately prevalent in regions with racial and ethnic minorities [59, 60]. Higher mortality rates in minority groups may also be a consequence of factors such as reduced access to insurance as well as implicit biases by healthcare providers [61]. As pointed out by Aggarwal et al., [62] lack of health insurance as well as inadequate coverage disproportionately affect racial minorities, with an uninsured prevalence of 15% among NH Black adults. Although, even when health insurance is provided through Medicaid, it is not enough to improve healthcare outcomes. Many studies have noted that medication adherence, a key factor in mitigating mortality due to hypertension and obesity, tends to be lower among minorities [63]. Thus, despite many advancements made in both the non-pharmacological and pharmacological management of hypertension and obesity, regrettably mortality rates due to both comorbid diseases have only risen disproportionately in the disenfranchised.

While mortality rates ascribed to obesity and hypertension rose in both non-metropolitan and metropolitan areas, consistently higher mortality rates were observed in non-metropolitan regions of the country. Reinforcing this view, we observed some of the lowest mortality rates in states like Connecticut, Alabama, Virginia, Massachusetts, and Missouri, all of whom are composed of largely urban populations [64]. Further echoing this sentiment, we observed some of the highest mortality rates among rural states directly adjacent to those with the lowest rates—including Vermont, bordering Massachusetts, West Virginia, bordering Virginia, Oklahoma, bordering Missouri, and Mississippi, bordering Alabama— underscoring the hypothesis that rurality itself may be a predictor of poor health outcomes. Declining health among America’s non-metropolitan areas appears to be a multi-faceted issue. For instance, such a trend could possibly reflect the well documented phenomenon that typically, non-metropolitan regions of the country tend to demonstrate worse health outcomes because of the lack of access to healthcare facilities [65] decreased primary care physician density [66], and reduced capacities of rural health systems [55, 67]. To further compound this issue, rural communities often face limitations in regards to access to health-related information, whether it may stem from media bias or reduced physician availability [68]. Such barriers result in poor health literacy, underutilization of healthcare, as well as reduced positive health behaviors in the community due to lack of awareness [69]. Finally, studies propose even if America’s rural population has awareness regarding healthy food choices and lifestyle factors to reduce hypertension and obesity related mortality, they do not have the ability to implement such lifestyle changes due to challenges associated with food insecurity [38]; Helmick et al., [70] found that food insecurity was responsible for up to a 77% increased risk of hypertension in rural communities.

However, among the states with the highest mortality rates, it is pertinent to mention that the majority were not only predominantly rural, like Vermont [64] but also located in the South, including Oklahoma, West Virginia, and Mississippi [71]. Conceivably, regional differences further exacerbate rural-urban disparities in this cohort. Myers et al. argues that perhaps obesity in the South is structurally different from obesity in the rest of the country, due to deeply embedded cultural factors [72] including perceptions and norms regarding dietary patterns and ideal body-weight unique to such a population. The notorious “Southern Diet Score,” [73] characterized by fried processed foods and sugar sweetened beverages, has been implicated in several studies as a large contributory factor in weight gain, obesity and hypertension among US adults in the South [74, 75]. Although, perhaps higher hypertension and obesity related mortality in the South is not independent of racial and socioeconomic divides. One study found that county level characteristics determining obesity rates can be attributed to higher density of African American populations in the South, who are known to face barriers in attaining healthful lifestyles [72]. As discussed previously, minority groups including those of low socioeconomic standing and people of color, in both urban and rural regions, are known to face inequalities in obesity due to factors such as walkable communities, and food deserts [76, 77]. Further compounding this issue, many studies have noted a higher concentration of fast-food chain restaurants in poorer neighborhoods [77]. Consequently, we cannot think of the higher mortality rates in people of color, in rural regions, or in southern regions as independent trends. Policies aimed at reducing the mortality burned due to hypertension and obesity therefore must be cognizant of the fact that these are not problems that exist individually and independently, rather represent a wider range of concerns that act in concert to amplify each other. There is no blanket solution— targeted interventions must be developed to address critical region-specific concerns that account for such demographic divides.

### Limitations

Despite our robust findings, the study has several limitations. Given the specificity of our results to the population of the United States, we cannot generalize our findings to other countries or regions. Our analysis lacked information regarding other co-morbid conditions and lifestyle factors that may potentially have a significant impact on obesity and hypertension related mortality. Therefore, we are unable to assess the influence of potential confounders like socioeconomic status, healthcare access, or comorbidities due to unavailability of data regarding them on the CDC WONDER database. Moreover, our analysis is limited by its reliance on ICD-10 codes and may be subject to misclassification. Furthermore, the retrieved death certificates do not report specified data regarding the control of hypertension. Therefore, we are unable to differentiate those individuals with controlled vs. uncontrolled hypertension or the relative impact of hypertension control on death.

### Future directions and conclusion

Despite decades of preventive efforts and therapeutic advances, obesity- and hypertension-related mortality in U.S. adults has climbed relentlessly, disproportionately affecting men, racial minorities, and rural communities. These findings underscore the need for culturally tailored, equity-focused public health strategies and health system reforms to reverse alarming trends and close persistent demographic gaps.

## Supplementary Information

Supplementary Material 1.

## Data Availability

Data is provided within the manuscript or supplementary information files.

## Abbreviations

AAPC: Average Annual Percent Change
APC: Annual Percent Change
AAMR: Age-Adjusted Mortality Rate
CDC: Centers for Disease Control and Prevention
CI: Confidence Interval
CMR: Crude Mortality Rate
CVD: Cardiovascular Disease
HTN: Hypertension
ICD-10: International Classification of Diseases, 10th Revision
NCHS: National Center for Health Statistics
NH: Non-Hispanic
STROBE: Strengthening the Reporting of Observational Studies in Epidemiology
US: United States
WONDER: Wide-Ranging Online Data for Epidemiologic Research
