# Supplementary material for: Concomitant mortality trends due to obesity and hypertension in the U.S.: a 20-year retrospective analysis of the CDC WONDER database
Source: BMC Cardiovasc Disord. 2025 Jul 7;25:496. doi: 10.1186/s12872-025-04909-z (PMC12232563; doi:10.1186/s12872-025-04909-z)

**Supplementary Table 1:** *Age-Adjusted Mortality Rates (AAMR) from Obesity and Hypertension in the US by* ***Gender*** *(2000–2019).*

| Gender | Year | Age Adjusted Rate | Age Adjusted Rate Lower 95% Confidence Interval | Age Adjusted Rate Upper 95% Confidence Interval | Age Adjusted Rate Standard Error |
| --- | --- | --- | --- | --- | --- |
| Female | 2000 | 2.5266 | 2.4266 | 2.6266 | 0.051 |
| Female | 2001 | 2.5508 | 2.4514 | 2.6503 | 0.0508 |
| Female | 2002 | 2.9254 | 2.8199 | 3.0308 | 0.0538 |
| Female | 2003 | 3.3051 | 3.1934 | 3.4168 | 0.057 |
| Female | 2004 | 3.4436 | 3.3303 | 3.5569 | 0.0578 |
| Female | 2005 | 3.6852 | 3.5692 | 3.8012 | 0.0592 |
| Female | 2006 | 3.7926 | 3.6761 | 3.9092 | 0.0595 |
| Female | 2007 | 3.9697 | 3.8509 | 4.0886 | 0.0606 |
| Female | 2008 | 4.2446 | 4.123 | 4.3663 | 0.0621 |
| Female | 2009 | 4.5726 | 4.4464 | 4.6988 | 0.0644 |
| Female | 2010 | 4.7253 | 4.5981 | 4.8524 | 0.0649 |
| Female | 2011 | 5.1617 | 5.0297 | 5.2936 | 0.0673 |
| Female | 2012 | 5.5801 | 5.4434 | 5.7167 | 0.0697 |
| Female | 2013 | 5.8476 | 5.7084 | 5.9868 | 0.071 |
| Female | 2014 | 6.1036 | 5.9626 | 6.2445 | 0.0719 |
| Female | 2015 | 6.5196 | 6.3745 | 6.6647 | 0.074 |
| Female | 2016 | 6.8514 | 6.7035 | 6.9992 | 0.0754 |
| Female | 2017 | 6.9167 | 6.7692 | 7.0643 | 0.0753 |
| Female | 2018 | 7.1683 | 7.019 | 7.3175 | 0.0762 |
| Female | 2019 | 7.5589 | 7.4064 | 7.7115 | 0.0778 |
| Male | 2000 | 2.5781 | 2.4692 | 2.6871 | 0.0556 |
| Male | 2001 | 2.6653 | 2.5557 | 2.7749 | 0.0559 |
| Male | 2002 | 3.1579 | 3.0397 | 3.2761 | 0.0603 |
| Male | 2003 | 3.5421 | 3.4186 | 3.6657 | 0.0631 |
| Male | 2004 | 3.8667 | 3.7388 | 3.9946 | 0.0653 |
| Male | 2005 | 4.2144 | 4.0823 | 4.3466 | 0.0674 |
| Male | 2006 | 4.4526 | 4.318 | 4.5872 | 0.0687 |
| Male | 2007 | 4.8895 | 4.7493 | 5.0297 | 0.0715 |
| Male | 2008 | 5.2381 | 5.0942 | 5.382 | 0.0734 |
| Male | 2009 | 5.8588 | 5.7084 | 6.0092 | 0.0767 |
| Male | 2010 | 6.2634 | 6.1082 | 6.4186 | 0.0792 |
| Male | 2011 | 6.8801 | 6.7185 | 7.0417 | 0.0825 |
| Male | 2012 | 7.5021 | 7.3349 | 7.6693 | 0.0853 |
| Male | 2013 | 7.9298 | 7.7589 | 8.1007 | 0.0872 |
| Male | 2014 | 8.7178 | 8.5393 | 8.8963 | 0.0911 |
| Male | 2015 | 9.3142 | 9.1318 | 9.4966 | 0.0931 |
| Male | 2016 | 9.7771 | 9.5905 | 9.9637 | 0.0952 |
| Male | 2017 | 10.3281 | 10.1381 | 10.5181 | 0.0969 |
| Male | 2018 | 11.1045 | 10.9087 | 11.3003 | 0.0999 |
| Male | 2019 | 11.7789 | 11.5781 | 11.9797 | 0.1025 |
|  |  |  |  |  |  |
|  |  |  |  |  |  |

**Supplementary Figure 1:** *Age-adjusted Mortality Rates’ Annual Percentage Change (APC) from Obesity and Hypertension in the US by* ***Gender*** *(2000–2019).*


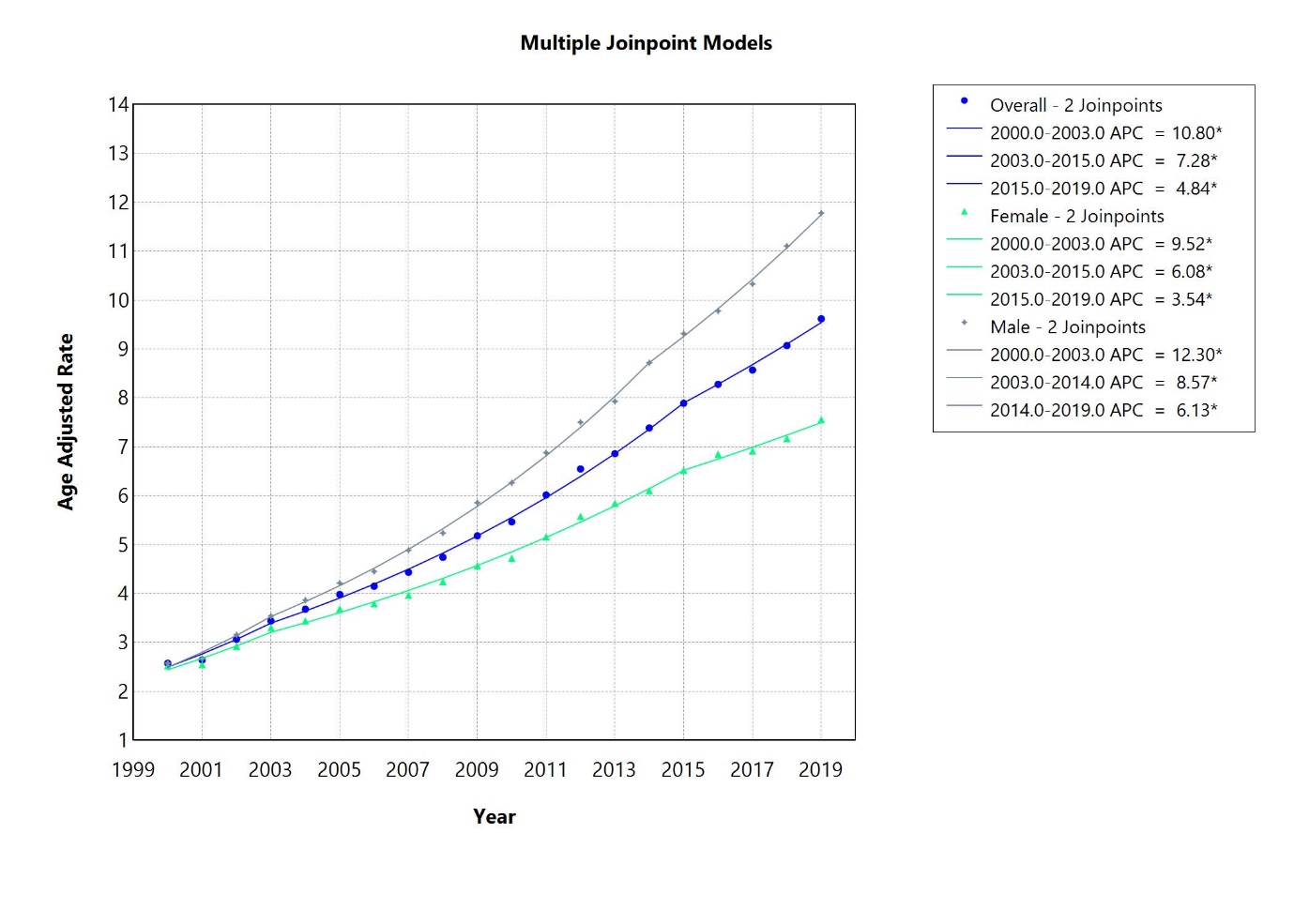


**Supplementary Table 2:** *Age-Adjusted Mortality Rates (AAMR) from Obesity and Hypertension in the US by* ***Race*** *(2000–2019).*

| Year | Age-Adjusted Mortality Rate | | | | |
| --- | --- | --- | --- | --- | --- |
|  | **American Indian or Alaska Native** | **Asian or Pacific Islander** | **Black or African American** | **White** | **Hispanic or Latino** |
| 2000 | 2.352 | 0.472 | 5.8589 | 2.2754 | 1.6339 |
| 2001 | 2.1943 | 0.2671 | 6.0743 | 2.276 | 1.7655 |
| 2002 | 3.2138 | 0.5034 | 6.9884 | 2.6598 | 1.9474 |
| 2003 | 3.4592 | 0.6489 | 7.5683 | 3.0657 | 2.5278 |
| 2004 | 3.2441 | 0.6201 | 8.355 | 3.2088 | 2.4131 |
| 2005 | 3.993 | 0.9132 | 8.4353 | 3.5579 | 2.361 |
| 2006 | 5.0872 | 0.7941 | 8.5782 | 3.7198 | 2.9623 |
| 2007 | 4.0087 | 1.0619 | 8.968 | 3.9936 | 3.0675 |
| 2008 | 4.6478 | 1.3555 | 9.3141 | 4.3268 | 3.173 |
| 2009 | 7.1957 | 0.9531 | 10.23 | 4.7274 | 3.916 |
| 2010 | 6.2198 | 1.2205 | 10.5346 | 4.9876 | 3.9897 |
| 2011 | 5.7663 | 1.3325 | 11.1085 | 5.5428 | 4.3494 |
| 2012 | 7.2782 | 1.3244 | 12.0988 | 6.0274 | 4.522 |
| 2013 | 7.4591 | 1.1683 | 12.6306 | 6.3738 | 5.0657 |
| 2014 | 9.0548 | 1.553 | 13.1797 | 6.877 | 5.1849 |
| 2015 | 7.4264 | 1.9633 | 14.2718 | 7.3031 | 5.3803 |
| 2016 | 9.6899 | 1.6131 | 14.8787 | 7.6987 | 5.7508 |
| 2017 | 8.8822 | 1.9072 | 14.7587 | 8.0456 | 5.842 |
| 2018 | 9.9976 | 1.9815 | 15.6508 | 8.5349 | 5.904 |
| 2019 | 11.1966 | 2.0345 | 16.6469 | 8.9968 | 6.3345 |

**Supplementary Figure 2:** *Age-adjusted Mortality Rates’ Annual Percentage Change (APC) from Obesity and Hypertension in the US by* ***Race*** *(2000–2019).*


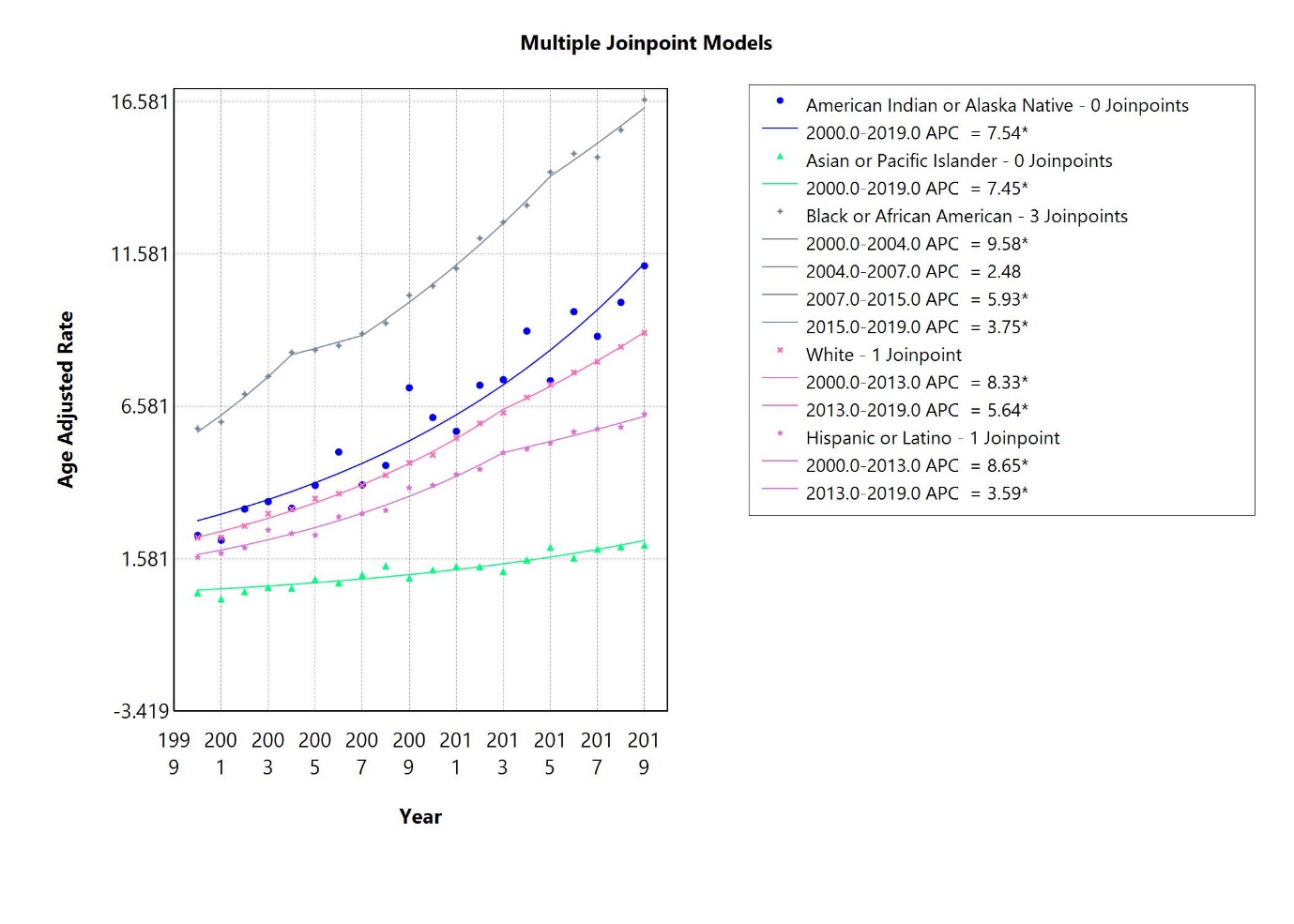


**Supplementary Table 3:** *Age-Adjusted Mortality Rates from Obesity and Hypertension in the US by* ***Metropolitan Status*** *(2000–2019).*

| Urbanization | Year | Deaths | Age Adjusted Rate | Age Adjusted Rate Lower 95% Confidence Interval | Age Adjusted Rate Upper 95% Confidence Interval | Age Adjusted Rate Standard Error |
| --- | --- | --- | --- | --- | --- | --- |
| Metropolitan | 2000 | 3801 | 2.5765 | 2.4946 | 2.6585 | 0.0418 |
| Metropolitan | 2001 | 3979 | 2.6148 | 2.5335 | 2.6961 | 0.0415 |
| Metropolitan | 2002 | 4669 | 3.0041 | 2.9179 | 3.0904 | 0.044 |
| Metropolitan | 2003 | 5420 | 3.4317 | 3.3402 | 3.5233 | 0.0467 |
| Metropolitan | 2004 | 5833 | 3.6128 | 3.5198 | 3.7058 | 0.0475 |
| Metropolitan | 2005 | 6399 | 3.8489 | 3.7542 | 3.9436 | 0.0483 |
| Metropolitan | 2006 | 6841 | 4.0663 | 3.9694 | 4.1632 | 0.0494 |
| Metropolitan | 2007 | 7547 | 4.3701 | 4.2709 | 4.4694 | 0.0506 |
| Metropolitan | 2008 | 8136 | 4.6382 | 4.5366 | 4.7397 | 0.0518 |
| Metropolitan | 2009 | 9140 | 5.1043 | 4.9986 | 5.21 | 0.0539 |
| Metropolitan | 2010 | 9777 | 5.3718 | 5.2642 | 5.4795 | 0.0549 |
| Metropolitan | 2011 | 10900 | 5.895 | 5.7827 | 6.0073 | 0.0573 |
| Metropolitan | 2012 | 12042 | 6.3721 | 6.2566 | 6.4877 | 0.0589 |
| Metropolitan | 2013 | 12910 | 6.7208 | 6.6029 | 6.8387 | 0.0602 |
| Metropolitan | 2014 | 14048 | 7.1924 | 7.0712 | 7.3136 | 0.0618 |
| Metropolitan | 2015 | 15324 | 7.6586 | 7.5349 | 7.7823 | 0.0631 |
| Metropolitan | 2016 | 16291 | 8.0619 | 7.9352 | 8.1885 | 0.0646 |
| Metropolitan | 2017 | 17137 | 8.3127 | 8.1852 | 8.4401 | 0.065 |
| Metropolitan | 2018 | 18365 | 8.7867 | 8.6565 | 8.9169 | 0.0664 |
| Metropolitan | 2019 | 19490 | 9.2057 | 9.0731 | 9.3383 | 0.0676 |
| Non-metropolitan | 2000 | 844 | 2.664 | 2.4835 | 2.8446 | 0.0921 |
| Non-metropolitan | 2001 | 856 | 2.6867 | 2.5058 | 2.8677 | 0.0923 |
| Non-metropolitan | 2002 | 1072 | 3.3352 | 3.1341 | 3.5364 | 0.1026 |
| Non-metropolitan | 2003 | 1167 | 3.5579 | 3.3519 | 3.7638 | 0.1051 |
| Non-metropolitan | 2004 | 1311 | 3.9614 | 3.7449 | 4.1779 | 0.1104 |
| Non-metropolitan | 2005 | 1507 | 4.4679 | 4.2395 | 4.6963 | 0.1165 |
| Non-metropolitan | 2006 | 1571 | 4.5756 | 4.3461 | 4.8051 | 0.1171 |
| Non-metropolitan | 2007 | 1599 | 4.6365 | 4.4051 | 4.8679 | 0.1181 |
| Non-metropolitan | 2008 | 1852 | 5.3058 | 5.0591 | 5.5524 | 0.1259 |
| Non-metropolitan | 2009 | 2028 | 5.705 | 5.4509 | 5.9592 | 0.1297 |
| Non-metropolitan | 2010 | 2131 | 5.9489 | 5.6891 | 6.2086 | 0.1325 |
| Non-metropolitan | 2011 | 2395 | 6.6849 | 6.4086 | 6.9612 | 0.141 |
| Non-metropolitan | 2012 | 2648 | 7.3815 | 7.0898 | 7.6732 | 0.1488 |
| Non-metropolitan | 2013 | 2777 | 7.6761 | 7.3794 | 7.9728 | 0.1514 |
| Non-metropolitan | 2014 | 3083 | 8.4274 | 8.117 | 8.7378 | 0.1584 |
| Non-metropolitan | 2015 | 3321 | 9.0171 | 8.6961 | 9.3381 | 0.1638 |
| Non-metropolitan | 2016 | 3507 | 9.4905 | 9.1601 | 9.821 | 0.1686 |
| Non-metropolitan | 2017 | 3774 | 10.0552 | 9.7176 | 10.3928 | 0.1722 |
| Non-metropolitan | 2018 | 4076 | 10.7168 | 10.3691 | 11.0645 | 0.1774 |
| Non-metropolitan | 2019 | 4548 | 11.9117 | 11.5448 | 12.2787 | 0.1872 |

**Supplementary Figure 3:** Age-adjusted Mortality Rates’ Annual Percentage Change (APC) from Obesity and Hypertension in the US by **Metropolitan Status** (2000–2019).


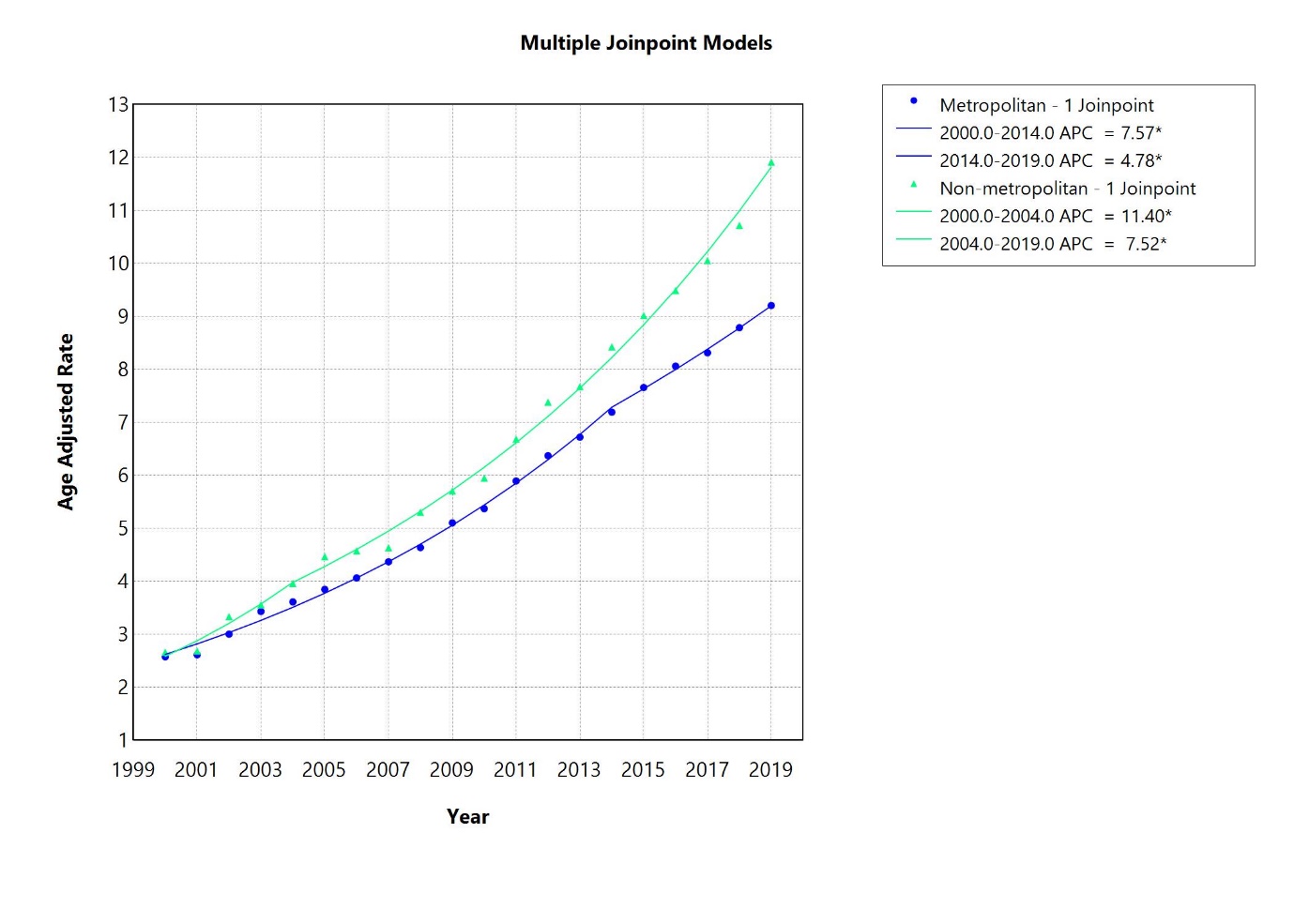


**Supplementary Table 4:** *Age-Adjusted Mortality Rates from Obesity and Hypertension-only in the US (2000–2019).*

| **UCOD** | **Year** | **Deaths** | **Population** | **Age Adjusted Rate** | **Age Adjusted Rate Lower 95% Confidence Interval** | **Age Adjusted Rate Upper 95% Confidence Interval** | **Age Adjusted Rate Standard Error** |
| --- | --- | --- | --- | --- | --- | --- | --- |
| Hypertension | 2000 | 44551 | 181984640 | 24.9613 | 24.7295 | 25.1931 | 0.1183 |
| Hypertension | 2001 | 46696 | 184305128 | 25.7272 | 25.4938 | 25.9605 | 0.1191 |
| Hypertension | 2002 | 49638 | 186208028 | 26.9019 | 26.6652 | 27.1387 | 0.1208 |
| Hypertension | 2003 | 53306 | 188090429 | 28.3729 | 28.1319 | 28.614 | 0.123 |
| Hypertension | 2004 | 54621 | 190205384 | 28.6095 | 28.3693 | 28.8497 | 0.1226 |
| Hypertension | 2005 | 57280 | 192551384 | 29.3803 | 29.1392 | 29.6213 | 0.123 |
| Hypertension | 2006 | 56473 | 195019359 | 28.2885 | 28.0546 | 28.5224 | 0.1193 |
| Hypertension | 2007 | 57653 | 197403777 | 28.2237 | 27.9924 | 28.4549 | 0.118 |
| Hypertension | 2008 | 60905 | 199795090 | 29.204 | 28.9709 | 29.437 | 0.1189 |
| Hypertension | 2009 | 61659 | 202107016 | 28.9073 | 28.6778 | 29.1369 | 0.1171 |
| Hypertension | 2010 | 63034 | 203891983 | 29.0627 | 28.8341 | 29.2912 | 0.1166 |
| Hypertension | 2011 | 65038 | 206592936 | 29.126 | 28.9001 | 29.3519 | 0.1153 |
| Hypertension | 2012 | 68030 | 208826037 | 29.7288 | 29.5031 | 29.9545 | 0.1152 |
| Hypertension | 2013 | 71864 | 211085314 | 30.7448 | 30.5174 | 30.9721 | 0.116 |
| Hypertension | 2014 | 73263 | 213809280 | 30.7481 | 30.5226 | 30.9736 | 0.115 |
| Hypertension | 2015 | 78786 | 216553817 | 32.3848 | 32.1555 | 32.6141 | 0.117 |
| Hypertension | 2016 | 82641 | 218641417 | 33.3445 | 33.1138 | 33.5752 | 0.1177 |
| Hypertension | 2017 | 89999 | 221447331 | 35.5465 | 35.3109 | 35.7821 | 0.1202 |
| Hypertension | 2018 | 95789 | 223311190 | 37.119 | 36.8806 | 37.3573 | 0.1216 |
| Hypertension | 2019 | 101994 | 224981167 | 38.8017 | 38.5603 | 39.0432 | 0.1232 |
| Obesity | 2000 | 2938 | 181984640 | 1.6202 | 1.5616 | 1.6788 | 0.0299 |
| Obesity | 2001 | 3076 | 184305128 | 1.6723 | 1.6131 | 1.7314 | 0.0302 |
| Obesity | 2002 | 3613 | 186208028 | 1.9421 | 1.8787 | 2.0055 | 0.0323 |
| Obesity | 2003 | 3944 | 188090429 | 2.0465 | 1.9825 | 2.1104 | 0.0326 |
| Obesity | 2004 | 4144 | 190205384 | 2.1257 | 2.0608 | 2.1906 | 0.0331 |
| Obesity | 2005 | 4569 | 192551384 | 2.2986 | 2.2316 | 2.3655 | 0.0342 |
| Obesity | 2006 | 4664 | 195019359 | 2.3066 | 2.2401 | 2.3732 | 0.034 |
| Obesity | 2007 | 4784 | 197403777 | 2.3635 | 2.296 | 2.431 | 0.0344 |
| Obesity | 2008 | 4945 | 199795090 | 2.3882 | 2.3211 | 2.4553 | 0.0342 |
| Obesity | 2009 | 5340 | 202107016 | 2.507 | 2.439 | 2.5751 | 0.0347 |
| Obesity | 2010 | 5467 | 203891983 | 2.5515 | 2.4828 | 2.6201 | 0.035 |
| Obesity | 2011 | 5879 | 206592936 | 2.7075 | 2.6372 | 2.7778 | 0.0359 |
| Obesity | 2012 | 6115 | 208826037 | 2.7579 | 2.6875 | 2.8283 | 0.0359 |
| Obesity | 2013 | 6369 | 211085314 | 2.8271 | 2.7564 | 2.8977 | 0.036 |
| Obesity | 2014 | 6800 | 213809280 | 2.9951 | 2.9224 | 3.0678 | 0.0371 |
| Obesity | 2015 | 7350 | 216553817 | 3.1946 | 3.1198 | 3.2693 | 0.0381 |
| Obesity | 2016 | 7648 | 218641417 | 3.2949 | 3.2191 | 3.3707 | 0.0387 |
| Obesity | 2017 | 7668 | 221447331 | 3.22 | 3.1459 | 3.294 | 0.0378 |
| Obesity | 2018 | 7834 | 223311190 | 3.2381 | 3.1643 | 3.3119 | 0.0376 |
| Obesity | 2019 | 8286 | 224981167 | 3.3851 | 3.31 | 3.4603 | 0.0384 |

**Supplementary Figure 4:** Age-adjusted Mortality Rates’ Annual Percentage Change (APC) from Obesity and Hypertension-only in the US (2000–2019).


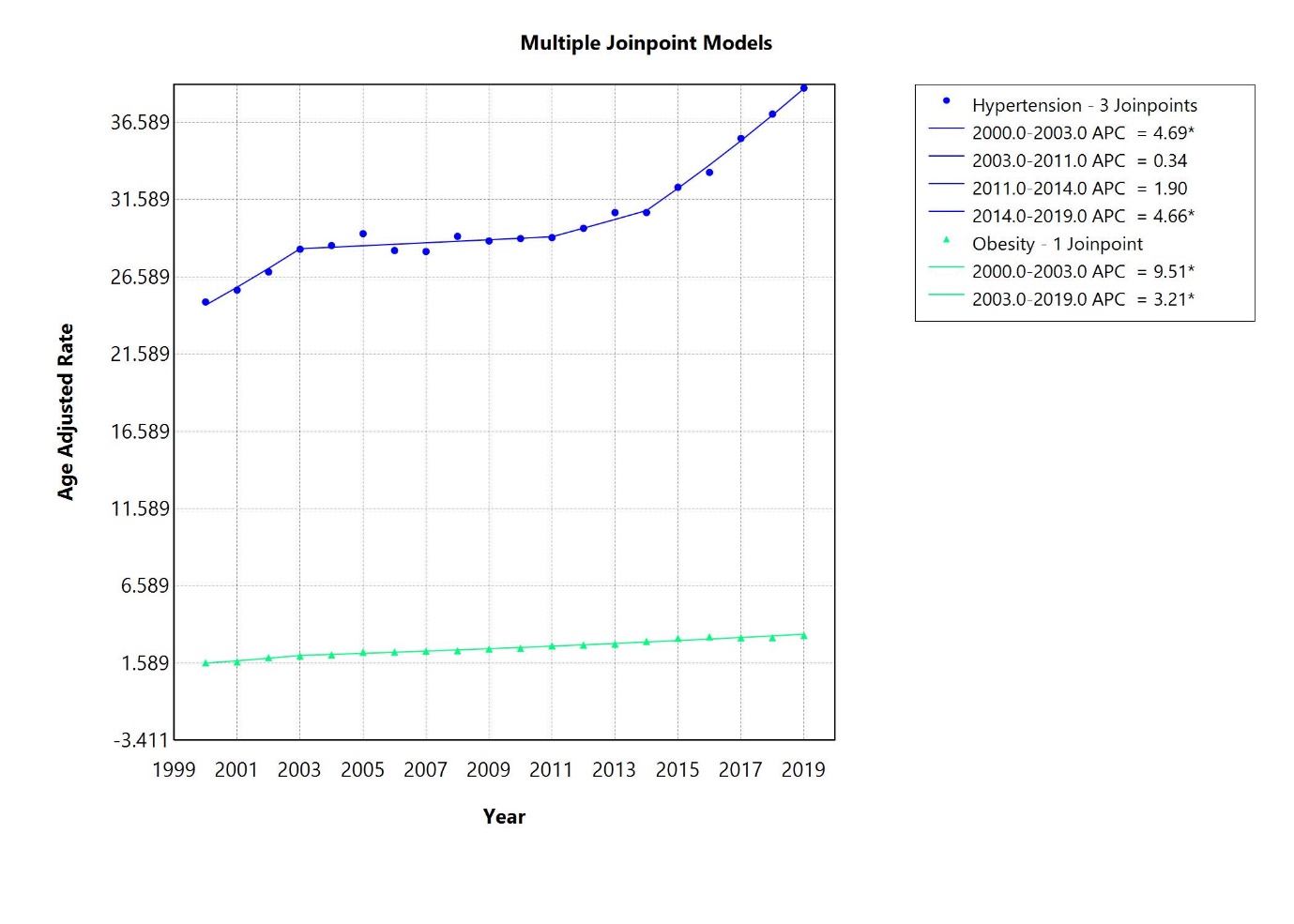

Supplement: Supplementary file 1 — Supplementary Material 1. [file 12872_2025_4909_MOESM1_ESM.docx]
